# Influence of peritoneal carcinomatosis on perioperative outcome in palliative gastric bypass for malignant gastric outlet obstruction - a retrospective cohort study

**DOI:** 10.1186/s12957-020-1803-5

**Published:** 2020-01-31

**Authors:** Jan Bednarsch, Zoltan Czigany, Daniel Heise, Henning Zimmermann, Joerg Boecker, Tom Florian Ulmer, Ulf Peter Neumann, Christian Klink

**Affiliations:** 1 Department of Surgery and Transplantation, University Hospital RWTH Aachen, Pauwelsstrasse 30, 52074 Aachen, Germany; 2 Department of Internal Medicine III, University Hospital RWTH Aachen, Pauwelsstrasse 30, 52074 Aachen, Germany; 3 Department of Surgery, Maastricht University Medical Centre (MUMC), P. Debyelaan 25, 6229 HX, Maastricht, Netherlands

**Keywords:** Gastric outlet obstruction, Gastrojejujnostomy, Peritoneal carcinomatosis

## Abstract

**Background:**

Malignant gastric outlet obstruction (GOO) is commonly associated with the presence of peritoneal carcinomatosis (PC) and preferably treated by surgical gastrojejunostomy (GJJ) in patients with good performance. Here, we aim to investigate the role of PC as a risk factor for perioperative morbidity and mortality in patients with GOO undergoing GJJ.

**Methods:**

Perioperative data of 72 patients with malignant GOO who underwent palliative GJJ at our institution between 2010 and 2019 were collected within an institutional database.

To compare perioperative outcomes of patients with and without PC, extensive group analyses were carried out.

**Results:**

A set of 39 (54.2%) patients was histologically diagnosed with concomitant PC while the remaining 33 (45.8%) patients showed no clinical signs of PC. In-house mortality due to surgical complications was significantly higher in patients with PC (9/39, 23.1%) than in patients without PC (2/33, 6.1%, *p* = .046). Considerable differences were observed in terms of surgical complications such as anastomotic leakage rates (2.8% vs. 0%, *p* = .187), delayed gastric emptying (33.3% vs. 15.2%, *p* = .076), paralytic ileus (23.1% vs. 9.1%, *p* = .113), and pneumonia (17.9% vs. 12.1%, *p* = .493) without reaching the level of statistical significance.

**Conclusions:**

PC is an important predictor of perioperative morbidity and mortality patients undergoing GJJ for malignant GOO.

## Background

Malignant gastric outlet obstruction (GOO) is a common complication of advanced gastric, pancreatic, or biliary tumors as well as peritoneal carcinomatosis (PC) of various origin. The presence of GOO in patients with advanced cancer is associated with dehydration and malnutrition and significantly impacts the quality of life (QoL) due to symptoms like nausea, pain, weight loss, and recurrent vomiting [1, 2]. This deterioration of the patient’s general condition and performance often results in interruption of systemic chemotherapy and requires rapid treatment to recover and continue medical therapy [3].

Surgical gastrojejunostomy (GJJ) has been considered as the mainstay of treatment for GOO [4]. However, in the last decade, endoscopic placement of a duodenal stent (DS) is becoming increasingly popular due to its simplicity and minimally invasive nature, leading to a faster recovery of oral intake and shorter hospital stay [5]. While DS is associated with better short-term outcomes, GJJ is preferable in patients with a longer life expectancy and good performance status since stent-related complications (e.g., reocclusion and stent migration) do not occur and reinterventions are usually not necessary after GJJ [6]. Thus, GJJ is usually performed in case of GOO if the patient is intraoperatively diagnosed with PC or the primary tumor related to GOO is intraoperatively considered as technically not resectable [7].

PC is a late stage manifestation of several gastrointestinal malignancies characterized by tumor dissemination across the peritoneal cavity and frequently observed in patients with advanced gastric, hepatobiliary, or pancreatic cancer [8–10]. Patients with PC commonly present with symptoms such as nausea, abdominal pain or weight loss, and cachexia as the disease progresses over time [11, 12]. Metachronous PC itself is also a prevalent cause of GOO in these patients.

Since PC represents an advanced stage of oncologic disease which is associated with malnutrition and impairment of the patients general condition, it seems plausible that the patients with PC are more prone to surgical complications and poor perioperative outcomes compared to individuals without PC [13]. Therefore, we here aim to investigate the role of PC as a risk factor for perioperative morbidity and mortality in patients with GOO undergoing GJJ.

## Methods

### Patients

Seventy-two (*n* = 72) consecutive patients with malignant gastric obstruction, who underwent palliative GJJ at the University Hospital RWTH Aachen (UH-RWTH) between 2010 and 2019, were included in this study. This retrospective study was conducted at the UH-RWTH in accordance with the requirements of the Institutional Review Board of the RWTH Aachen University, the current version of the Declaration of Helsinki, and the good clinical practice guidelines (ICH-GCP). Demographic characteristics are shown in Table 1.

**Table 1:** Patients’ characteristics

	Overall cohort (*n* = 72)	Peritoneal carcinomatosis vs. no peritoneal carcinomatosis (PC)		
		PC cohort (*n* = 39)	No PC cohort (*n* = 33)	*p* value
Demographics				
Sex, *n* (%)				.502
Male	38 (52.8)	22 (56.4)	16 (48.5)	
Female	34 (47.2)	17 (43.6)	17 (51.5)	
Age (years)	66 (58–74)	66 (57–74)	65 (59–74)	.591
BMI (kg/m^2^)	24 (21–26)	23 (21–26)	24 (21–27)	.635
ASA, *n* (%)				.654
I	0	0	0	
II	19 (26.4)	12 (30.8)	7 (21.2)	
III	45 (62.5)	23 (59.0)	22 (66.7)	
IV	8 (11.1)	4 (10.3)	4 (12.1)	
V	0		0	
Tumor characteristics				
Primary tumor, *n* (%)				.324
Pancreatic adenocarcinoma	42 (58.3)	18 (46.2)	24 (72.7)	
Cholangiocellular carcinoma	10 (13.9)	8 (20.5)	2 (6.1)	
Gastric adenocarcinoma	5 (6.9)	3 (7.7)	2 (6,1)	
Intestinal carcinoma	3 (4.2)	2 (5.1)	1 (3.0)	
Colorectal carcinoma	6 (8.3)	4 (10.3)	2 (6.1)	
Renal cell carcinoma	2 (2.8)	1 (2.6)	1 (3.0)	
Transitional cell carcinoma	1 (1.4)	1 (2.6)	0	
Mammarian carcinoma	2 (2.8)	2 (5.1)	0	
Ovarian cancer	1 (1.4)	0	1 (3.0)	
Synchronous/metachronous PC, *n* (%)				
Synchronous PC	n.a.	20 (51.3)	n.a.	
Metachronous PC	n.a.	19 (48.7)	n.a.	
Distant metastasis, *n* (%)				.132
Yes	24 (33.3)	10 (25.6)	14 (42.4)	
No	48 (66.7)	29 (74.4)	19 (57.6)	
Preoperative chemotherapy, *n* (%)				.776
Yes	10 (13.9)	5 (12.8)	5 (15.2)	
No	62 (86.1)	34 (87.2)	28 (84.8)	
Clinical chemistry				
Sodium (mmol/l)	139 (136–141)	139 (136–142)	138 (136–141)	.536
Hemoglobin (g/dl)	10.6 (9.3–12.1)	11.2 (9.0–12.7)	10.2 (9.4–11.7)	.235
Platelet count (nl)	242 (198–355)	250 (201–341)	215 (195–416)	.739
Total bilirubin (mg/dl)	0.6 (0.4–2.0)	0.59 (0.37–1.42)	0.76 (0.44–3.81)	.235
Prothrombin time (%)	87 (77–98)	89 (76–98)	85 (76–98)	.773
INR	1.1 (1.0–1.2)	1.1 (1.0–1.2)	1.1 (1.0–1.2)	.874
Albumin (g/l)	32 (29–39)	34 (28–38)	31 (28–39)	.394
Creatinine (mg/dl)	0.8 (0.6–1.0)	0.9 (0.6–1.1)	0.7 (0.6–0.9)	.230
CRP (mg/l)	29 (11–56)	25 (8–51)	31 (13–81)	.317
Operative data				
Laparoscopic surgery, *n* (%)	2 (2.8)	0	2 (6.1)	.119
Concomitant hepaticojejunostomy, *n* (%)	26 (36.1)	10 (25.6)	16 (48.5)	*.044*
Operative procedure, *n* (%)				.831
Antecolic approach	6 (8.3)	3 (7.7)	3 (9.1)	
Retrocolic approach	66 (91.7)	36 (92.3)	30 (90.9)	
Operation time (min)	170 (128–214)	160 (113–215)	192 (150–216)	.124
Intraoperative blood transfusion (U)	0	0	0 (0–2)	.817
Intraoperative fresh frozen plasma (U)	0	0	0	.108
Postoperative data				
Intensive care, days	1 (0–2)	1 (0–2)	1 (0–3)	.240
Hospitalization, days	13 (9–18)	13 (10–20)	12 (9–17)	.392
PBD*, *n* (%)	6 (13.0)	5 (17.2)	1 (5.9)	.270
Postoperative complications, *n* (%)				.381
No complications	23 (31.9)	13 (33.3)	10 (30.3)	
Clavien-Dindo I	8 (11.1)	4 (10.3)	4 (12.1)	
Clavien-Dindo II	20 (27.8)	8 (20.5)	12 (36.4)	
Clavien-Dindo IIIa	4 (5.6)	2 (5.1)	2 (6.1)	
Clavien-Dindo IIIb	5 (6.9)	2 (5.1)	3 (9.1)	
Clavien-Dindo IVa	1 (1.4)	1 (2.6)	0	
Clavien-Dindo IVb	0	0	0	
Clavien-Dindo V	11 (15.3)	9 (23.1)	2 (6.1)	
In-house mortality, *n* (%)	11 (15.3)	9 (23.1)	2 (6.1)	*.046*
Anastomotic stenosis, *n* (%)	2 (2.8)	1 (2.6)	1 (3)	.905
Anastomotic leakage, *n* (%)	2 (2.8)	2 (5.1)	0	.187
Delayed gastric emptying, *n* (%)	18 (25.0)	13 (33.3)	5 (15.2)	.076
Paralytic ileus, *n* (%)	12 (16.7)	9 (23.1)	3 (9.1)	.113
Pneumonia, *n* (%)	11 (15.3)	7 (17.9)	4 (12.1)	.493

Data presented as median and interquartile range if not noted otherwise. Categorical data were compared using the chi-squared test, Fisher’s exact test, or linear-by-linear association according to the scale and number of cases. Data derived from continuous variables of different groups were compared by the Mann-Whitney *U* test

*CRP* c-reactive protein, *INR* international normalized ratio, *PBD* percutaneous biliary drainage, *PC* peritoneal carcinomatosis

*Statistics are calculated for patients who did not undergo concomitant hepaticojejunostomy during the surgical procedure

### Clinical course and surgical technique

All patients who were referred for surgical treatment to our institution underwent a detailed clinical work-up. Every patient included in this study presented with obstructive gastric outlet syndrome due to a malignant stenosis and was discussed in an interdisciplinary tumor board. The assessment of the patients’ perioperative risk was based on the American Society of Anesthesiologists (ASA) score and the indication for surgery, and the selection of the operative procedure was made by an experienced visceral surgeon.

**Fig. 1:**
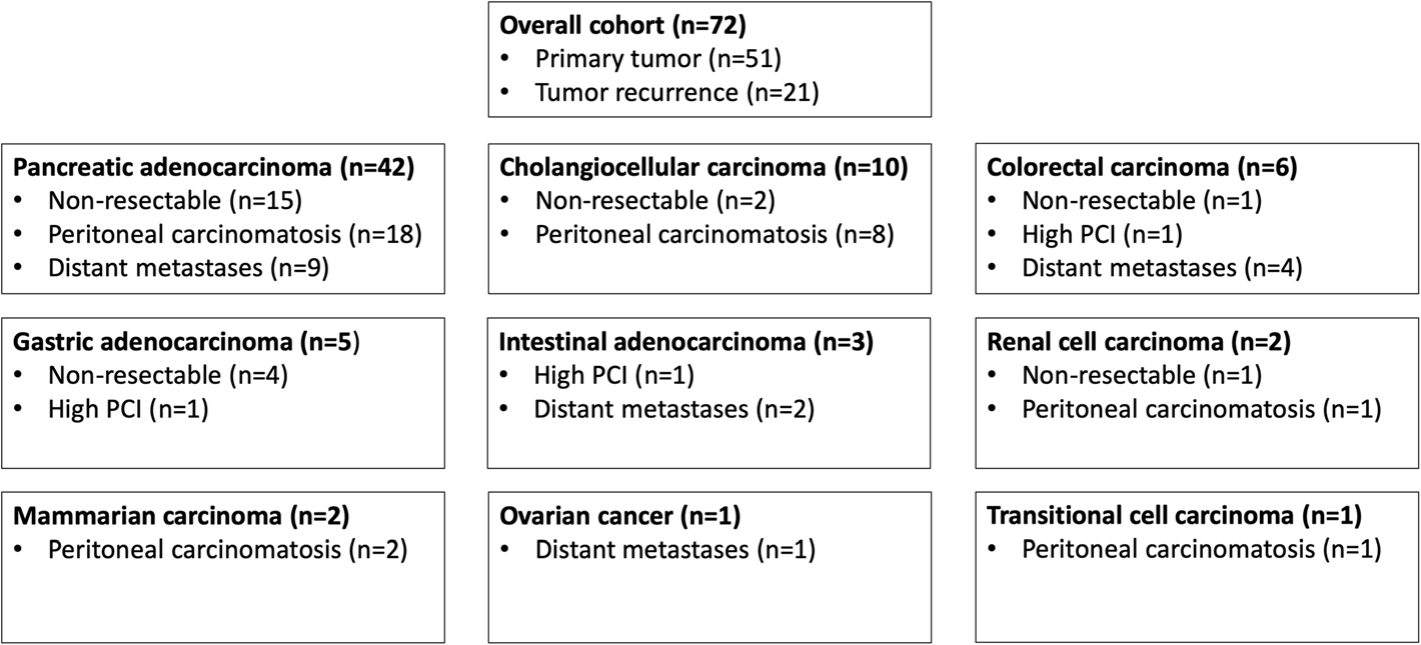
Overall cohort of patients undergoing gastric outlet obstruction. The overall cohorts comprised patients with pancreatic, cholangiocellular, gastric, intestinal, colorectal, renal cell, transitional cell, and mammarian carcinoma as well as ovarian cancer. Surgical gastrojejunostomy instead of curative surgery was considered in cases with technically non-resectable cancer (pancreatic, cholangiocellular, intestinal, colorectal, and renal cell carcinoma), distant metastases (pancreatic, cholangiocellular, gastric, and colorectal carcinoma and ovarian cancer), peritoneal carcinomatosis presenting with a high PCI in tumor entities qualifying for cytoreductive surgery and HIPEC (gastric, intestinal, and colorectal carcinoma) as well as cases presenting with peritoneal carcinomatosis tumor entities which preclude curative surgery (pancreatic, cholangiocellular, renal cell, transitional cell, and mammarian carcinoma). HIPEC, hyperthermic intraperitoneal chemotherapy; PCI, peritoneal carcinomatosis index

The majority of patients was diagnosed with an advanced tumor of hepatobiliary, pancreatic, duodenal, or gastric origin and underwent surgical exploration in curative intent. All patients presented with clinical symptoms of GOO preoperatively and showed signs of GOO in the preoperative cross-sectional imaging. If the local tumor was assessed as unresectable by the attending surgeon or if distant metastases (e.g., liver metastases or distant lymph nodes) and/or peritoneal carcinomatosis were diagnosed intraoperatively, a palliative gastrojejunostomy was carried out. In cases with significant biliary obstruction, an additional surgical hepaticojejunostomy was performed or it was resolved by percutaneous biliary drainage (PBD) during the postoperative course. The minority of patients underwent surgical exploration due to local recurrence of a previously resected malignant abdominal tumor and was also intraoperatively assessed as unresectable or showed distant metastases or PC. A detailed overview of the cohort and the particular surgical consideration for the GJJ is presented in Fig. 1.

Briefly, the surgical technique comprised an open hand-sewing technique with a side-to-side GJJ followed by a Roux-en-Y reconstruction with an end-to-side jejunojejunostomy. Whether a retrocolic or antecolic route for the GJJ was used was decided by the attending surgeon according to own preferences. The procedure was carried out laparoscopically in similar fashion with both anastomoses being realized by laparoscopic gastrointestinal staplers (Endo GIA, Medtronic, Dublin, Ireland).

The patients were treated postoperatively at a specialized intensive care unit or directly transferred to a normal postoperative ward based on the extent of the procedure and the individual preoperative medical condition. Parenteral nutrition was regularly used postoperatively if oral feeding could not be realized by the third postoperative day. No jejunostomy tubes were placed either intraoperatively or in the preoperative course in any patient of the cohort.

The presence of PC or distant metastases was intraoperatively indicated by fresh frozen sections and later confirmed by an experienced staff pathologist in the final pathological report in every case.

### Data collection

All study data including demographics, tumor characteristics, clinical chemistry, and operative and postoperative data of every patient were retrospectively collected within an institutional database. The postoperative course was reviewed for in-house mortality as well as complications and rated by the Clavien-Dindo Classification [14]. Every patient’s individual postoperative course was also assessed for specific surgical complications, e.g., anastomotic stenosis, anastomotic leakage, delayed gastric emptying (DGE), paralytic ileus, and pneumonia.

### Statistical analysis

The primary endpoint of this study was the incidence of in-house mortality in patients undergoing palliative GJJ with and without PC. The secondary endpoints were perioperative complications, duration of hospitalization, and ICU stay. Categorical data are presented as counts and percentages, compared using the chi-squared test, Fisher’s exact test, or linear-by-linear association according to the scale and number of cases. Data derived from continuous variables are presented as median and interquartile range and are analyzed by the Mann-Whitney *U* test. Associations between pre- and intraoperative variables and postoperative mortality were assessed by means of binary logistic regression. The level of significance was set to *p* < 0.05, and *p* values are given for two-sided testing. Analyses were performed using SPSS Statistics 24 (IBM Corp., Armonk, NY, USA).

## Results

### Patient cohort

A total of 72 patients underwent palliative GJJ due to malignant GOO at our institution from 2010 to 2019. A subset of 39 (54.2%) patients was histologically diagnosed with concomitant PC while the remaining 33 (45.8%) patients showed no clinical signs of PC.

### Preoperative, intraoperative, and postoperative data

The overall cohort comprised 38 (52.8%) male and 34 (47.2%) female patients with a median age of 66 years who were mostly assessed ASA III or higher (53/72, 73.6%). The majority of patients was diagnosed with pancreatic adenocarcinoma (42/72, 58.3%) followed by cholangiocellular carcinoma (10/72, 13.9%), colorectal cancer (6/72, 8.3%), and gastric adenocarcinoma (5/72, 6.9%). Only a subset of patients was treated with chemotherapy prior to surgery (10/72, 13.9%). No statistical difference was observed between patients with and without PC with respect to demographics and tumor characteristics, e.g., the primary diagnosis (*p* = .324), the presence of distant metastases (*p* = .132), or the utilization of preoperative chemotherapy (*p* = .776).

Laparoscopic surgery was seldomly applied in the cohort (2/72, 2.8%), and the gastrojejunostomy was mostly carried out in a retrocolic technique (66/72, 91.7%) with no difference between patients with and without PC (*p* = .831). Also, the median operating time showed no difference in patients with and without PC (160 min vs. 192 min, *p* = .124). However, an additional hepaticojejunostomy was more common in patients without PC (16/33, 48.5%) than in patients with PC (10/39, 25.6%, *p* = .044).

No difference was observed in the median duration of hospitalization (13 vs. 12 days, *p* = .392) and intensive care treatment (1 vs. 1 day, *p* = .240) between patients with and without PC. In-house mortality due to surgical complications was significantly higher in patients with PC (9/39, 23.1%) than in patients without PC (2/33, 6.1%, *p* = .046). Surgery-specific complications such as anastomotic leakage (2/39 (2.8%) vs. 0/33, (0%), *p* = .187), DGE (13/39 (33.3%) vs. 5/33 (15.2%), *p* = .076), paralytic ileus (9/39 (23.1%) vs. 3/33 (9.1%), *p* = .113), and pneumonia (7/39 (17.9%) vs. 4/33 (12.1%), *p* = .493) were tendentially higher in patients with PC than in patients without PC but did not achieve a statistically significant differences between the groups.

A univariate binary logistic regression was carried out for postoperative mortality including all available pre- and intraoperative variables for patients with PC (Table 2). Here, no statistical significance was observed between pre- and intraoperative characteristics and postoperative mortality.

**Table 2:** Univariable binary logistic regression of postoperative mortality in patients with peritoneal carcinomatosis undergoing gastrojejunostomy

Variables	In-house mortality	
	Exp (B)/HR	*p* value
Sex (male = 1)	0.571	.482
Age	1.072	.137
BMI	1.083	.349
ASA scale	3.309	.090
Primary tumor		
Pancreatic adenocarcinoma	1	
Other primary tumors	0.612	.521
Synchronous/metachronous PC		
Synchronous PC	0.438	.299
Metachronous PC	1	
Distant metastasis (no. = 1)	0.786	.789
Preoperative chemotherapy (no.=1)	2.571	.348
Sodium	0.996	.963
Hemoglobin	0.824	.350
Platelet count	1.000	.977
Total bilirubin	0.989	.954
Prothrombin time	0.973	.267
INR	20.409	.211
Albumin	0.946	.348
Creatinine	1.047	.967
CRP	1.009	.418
Laparoscopic surgery (no. = 1)	n.a.	n.a.
Concomitant hepaticojejunostomy (no. = 1)	0.292	.277
Operative procedure		
Antecolic approach	0.000	.999
Retrocolic approach	1	
Operation time (min)	1.000	.975
Intraoperative blood transfusion (no. = 1)	0.625	.687
Intraoperative fresh frozen plasma (no. = 1)	0.000	.999

Various parameters are tested for association with in-house mortality

*ASA* American Society of Anesthesiologists classification, *BMI* body mass index, *HR* ratio, *INR* international normalized ratio

More details regarding perioperative characteristics and group comparisons are presented in Table 1.

## Discussion

Since traditional imaging such as classic computed tomography (CT) and magnetic resonance imaging (MRI) as well as positron-emission tomography (PET) lacks sensitivity to preoperatively detect PC, patients with PC and GOO often undergo surgical exploration with curative intent [15–17]. However, if PC is subsequently diagnosed intraoperatively, a GJJ is often performed to treat GOO without delay.

Here, we aimed to evaluate the effects of PC on perioperative outcomes in patients who underwent GJJ for malignant GOO. Therefore, we investigated the incidence of postoperative complications in terms of total and surgery-specific complications in these particular patients. Our statistical group comparison showed that surgery-related in-house mortality was significantly higher in patients with PC (23.1%) than in patients without PC (6.1%). Furthermore, surgery-specific complications such as anastomotic leakage, DGE, paralytic ileus, and pneumonia appeared to be more common in the PC group than in the non-PC group.

The reason for this observation remains speculative. While the analyzed surgery-specific complications did not show statistical significance, each complication occurred numerically higher in the PC cohort which might have translated into the observed increased overall mortality rate after surgery. Another possible explanation might be subclinical intestinal obstruction distal to the GJJ which causes intestinal congestion and subsequently anastomotic problems or aspiration [18]. Also, malnutrition is a major problem in patients with PC and has been in the focus of research in the last decade resulting in its identification as an important predictor for postoperative complications in abdominal and extra-abdominal surgery [19–21]. In addition to malnutrition, tumor cachexia, a complex multifactorial condition that arises from a combination of metabolic alterations, systemic inflammation, and decreased appetite, is also a major concern in patients with increased tumor burden [22]. Tumor cachexia is directly associated with malnutrition and associated with impaired wound healing, increased risk for surgical complications, and impaired overall outcome [23, 24].

Our patients display an in-house mortality of 15.3% in the overall cohort with 23.1% mortality in patients with PC and 6.1% without PC. This mortality was based on anastomotic leakage in two individuals, while most of the other patients with fatal outcome presented with postoperative ileus and subsequently developed abdominal sepsis or severe pneumonia presumably due to aspiration. The reported in-house mortality might appear high for gastrointestinal surgery. However, reported mortality rates after GJJ for unresectable cancer range from 3 to 30% in literature [9, 25–29] supporting the validity of our data. Interestingly, the presence of PC as a risk factor for surgical outcome has not been directly investigated in previous reports. While previous literature, comprising various cohorts from the last couple of decades, focuses mostly on general outcome figures, the report of Poulsen et al. analyzed surgical outcomes of 165 patients of which 120 individuals presented with malignant and 45 individuals with benign GOO and conducted a detailed analysis of surgical morbidity and mortality [25]. In this paper, the observed 30-day mortality in case of malignant GOO was 29% with age, comorbidities, hypoalbuminemia, and hyponatremia being the major drivers of mortality in multivariate analysis. Unfortunately, the role of peritoneal carcinomatosis was also not investigated in this particular work, but it illustrates the importance of patient-related characteristics as a risk factor.

Of note in this context, the performance of a concomitant hepaticojejunostomy during the surgical procedure was significantly more frequent in our non-PC cohort (48.5%) than in our PC cohort (24.6%, *p* = .044). However, this additional procedure did obviously not translate into an increased risk for surgical complications. This particular finding, as well as the increase of mortality by peritoneal carcinomatosis as suggested by our data and the results of Poulsen et al., indicates a superiority of patient- and tumor-related characteristics over the surgical technique itself in the perioperative risk assessment for patients undergoing GJJ for malignant GOO [25].

While a mortality of 6.1% in patients without PC does encourage the utilization of palliative GJJ in these particular patients, the observed 23.1% mortality in patients with PC combined with the reported perioperative outcomes in the literature does demand a critical discussion of therapeutic alternatives in this subcohort. DS has evolved as a viable option for patients with malignant GOO and limited life expectancy [5]. DS is effective and less invasive compared with surgical GJJ but has been reported to be associated with higher rates of reintervention and recurrent obstructive symptoms reported [5, 30, 31]. However, it is debatable whether potential reinterventions on the long run are favorable compared to an increased perioperative mortality in patients with PC who per se have a reduced life expectancy [32, 33]. A recent report of Park et al. does investigate the role of PC and malignant ascites in gastric cancer [34]. The experienced group from South Korea observed a longer patency after GJJ compared to DS in patients with PC alone and in patients with PC and ascites as well as longer overall survival after GJJ in patients with PC and ascites. Anyhow, these superior results for GJJ might not be applicable to PC of other origins which are less responsive to palliative chemotherapy, e.g., hepatobiliary or pancreatic cancer. Another comparative therapy currently investigated is endoscopic ultrasound-guided GJJ (EUS-GJJ) which warrants further research [35]. From a pure surgical point of view, laparoscopic GJJ may provide an improvement over open GJJ but the currently available literature mainly consists of small case series or small case-control studies and 1 randomized trial comparing open and laparoscopic GJJ in only 24 patients [36, 37]. Given the limited data and the complexity of malignant GOO, more clinical evidence is needed to evaluate the potential benefits of the laparoscopic approach over conventional open surgery [36].

In summary, PC appears to be a major driver of complications and dismal outcome in GJJ. Our findings warrant further investigations to explore the exact role of PC on clinical outcomes in the palliative treatment of GOO. Unfortunately, we were not able to determine statistically significant predictors of impaired postoperative outcome in patients with PC within our small retrospective cohort. Thus, prospective clinical trials with adequate sample sizes stratifying data or treatment by the presence of PC are warranted.

Some obvious limitations of our retrospective study need to be considered when interpreting the results. Firstly, the data represent a retrospective single-center experience, reflecting our individual approach and surgical technique in GOO. Secondly, our cohort shows a considerable heterogeneity in terms of primary tumors and no separate analyses for each primary tumor were conducted. This might be of a major importance since some of the tumor entities are commonly known to be more aggressive, e.g., cholangiocellular carcinoma, than others. We particularly decided to include various primary tumors to gain sample size and strengthen our statistical analysis. Also, all demographics including the primary tumor and perioperative characteristics usually associated with perioperative outcome showed no statistical difference between patients with and without PC supporting the validity of our findings. However, it has to be taken into account that we are not able to correlate our findings with estimates of the severity of the carcinomatosis, e.g., peritoneal carcinomatosis index (PCI), due to the retrospective nature of the study. Thirdly, we were not able to report the outcome in benign GOO and are not able to compare our results with DS in case of malignant GOO as a comparative treatment. Anyhow, our aim was to investigate the role of PC as a potential risk factor for surgical complications in patients who undergo GJJ, since in clinical reality, GJJ is more common than DS in patients who are surgically explored for a curative-intent resection and are intraoperatively diagnosed with PC or considered to be technically not resectable.

## Conclusions

Notwithstanding the aforementioned limitations, we have identified PC as an important predictor of perioperative morbidity and mortality in a cohort of patients undergoing GJJ for malignant GOO. Larger multicentric studies are warranted to confirm and validate these findings.

## Data Availability

The datasets used and/or analyzed during the current study are available from the corresponding author on reasonable request.

## Abbreviations

ASA: American Society of Anesthesiologists
BMI: Body mass index
CRP: C-reactive protein
CT: Computed tomography
DGE: Delayed gastric emptying
DS: Duodenal stent
EBD: Endoscopic biliary drainage
EUS-GJJ: Endoscopic ultrasound-guided gastrojejunostomy
GJJ: Gastrojejunostomy
GOO: Gastric outlet obstruction
HIPEC: Hyperthermic intraperitoneal chemotherapy
MRI: Magnetic resonance imaging
PBD: Percutaneous biliary drainage
PC: Peritoneal carcinomatosis
PCI: Peritoneal carcinomatosis index
PET: Positron-emission tomography
QoL: Quality of life
RWTH: Rheinisch-Westfälische Technische Hochschule
UICC: Union for International Cancer Control
